# The impact of gestational diabetes on functional capacity of the infant gut microbiome is modest and transient

**DOI:** 10.1080/19490976.2024.2356277

**Published:** 2024-05-26

**Authors:** Ryan V. Chieu, Katharine Hamilton, Paul M. Ryan, Julia Copeland, Pauline W. Wang, Ravi Retnakaran, David S. Guttman, John Parkinson, Jill K. Hamilton

**Affiliations:** a Program in Molecular Medicine, Hospital for Sick Children Research Institute, Toronto, ON, Canada; b Department of Molecular Genetics, University of Toronto, Toronto, ON, Canada; c Division of Endocrinology, The Hospital for Sick Children, Toronto, ON, Canada; d Department of Pediatrics, University of Toronto, Toronto, ON, Canada; e Centre for the Analysis of Genome Evolution & Function, University of Toronto, Toronto, ON, Canada; f Department of Cell & Systems Biology, University of Toronto, Toronto, ON, Canada; g Leadership Sinai Centre for Diabetes, Mount Sinai Hospital, Toronto, ON, Canada; h Department of Biochemistry, University of Toronto, Toronto, ON, Canada

**Keywords:** Gestational diabetes mellitus, gut microbiome, metatranscriptomics, 16S rRNA sequence analysis, mode of delivery, sex, exclusive breastfeeding status

## Abstract

Gestational diabetes mellitus (GDM) is a metabolic complication that manifests as hyperglycemia during the later stages of pregnancy. In high resource settings, careful management of GDM limits risk to the pregnancy, and hyperglycemia typically resolves after birth. At the same time, previous studies have revealed that the gut microbiome of infants born to mothers who experienced GDM exhibit reduced diversity and reduction in the abundance of several key taxa, including *Lactobacillus*. What is not known is what the functional consequences of these changes might be. In this case control study, we applied 16S rRNA sequence surveys and metatranscriptomics to profile the gut microbiome of 30 twelve-month-old infants − 16 from mothers with GDM, 14 from mothers without – to examine the impact of GDM during pregnancy. Relative to the mode of delivery and sex of the infant, maternal GDM status had a limited impact on the structure and function of the developing microbiome. While GDM samples were associated with a decrease in alpha diversity, we observed no effect on beta diversity and no differentially abundant taxa. Further, while the mode of delivery and sex of infant affected the expression of multiple bacterial pathways, much of the impact of GDM status on the function of the infant microbiome appears to be lost by twelve months of age. These data may indicate that, while mode of delivery appears to impact function and diversity for longer than anticipated, GDM may not have persistent effects on the function nor composition of the infant gut microbiome.

## Introduction

The human gut microbiome is increasingly viewed as a key determinant of health, with evidence supporting links to an ever-increasing number of diseases from diabetes and obesity to depression.^1,2^ While the microbiome can exhibit dramatic changes over the course of an individual’s life, how it develops over the first three years has a critical impact on determining its future contributions to health, disease, and immune system maturation.^3–5^ Among the most impactful factors that contribute to this development are mode of delivery, use of antenatal or postpartum antibiotics, and diet. Initial colonization is largely driven by mode of delivery, with vaginal deliveries associated with the dominance of *Bifidobacterium, Lactobacillus* and *Bacteroides* ,^6^ species that experience reduced abundance in infants delivered through Caesarean-section (C-section). As an infant’s diet changes from primarily breast or formula milk feeding with the introduction of solids, the microbiome increases in complexity.^7^ While most studies have focused on the dynamics of community composition using 16S rDNA sequence surveys, our knowledge of functional changes associated with the microbiome at this critical stage of development is limited.

In addition to these impactful postnatal factors, the metabolic status of the mother during pregnancy has also been found to contribute to the formation of the infant microbiome^8^ and future health status.^9^ *In utero* exposure to maternal type 1 diabetes (T1D), and in particular poorly regulated T1D, has recently been associated with a distinct infant rectal and skin microbiomes as early as the first day of life.^10^ Gestational diabetes mellitus (GDM) is a relatively common metabolic derangement of pregnancy, occurring in 3–20% of pregnancies^11^ in which mothers exhibit hyperglycemia during the later stages of gestation.^12^ While maternal hyperglycemia is generally manageable with dietary changes, and in fewer cases exogenous insulin or metformin, and often resolves after birth, it can increase the risk of type 2 diabetes and other metabolic disorders in both the mother and offspring in later life.^13–15^ 16S rRNA gene surveys have shown that the gut microbiota of infants born to mothers with GDM exhibit a significant decrease in alpha diversity,^16,17^ together with a loss in abundance of several taxa, including *Lactobacillus* and *Flavonifractor*.^18^ A recent study evaluating the neonatal ear-skin microbiome following delivery found differences in infants born to women with type 1 diabetes compared to neonates born to control mothers. The composition of the neonatal ear-skin microbiome was related to maternal levels of HbA1c in first trimester in a beta-diversity analysis.^10^ While such dysbioses in infants have been postulated as contributory to the increased risk of cardiometabolic diseases, little is known concerning the functional implications of such microbiome shifts.

Whole microbiome RNA sequencing, or metatranscriptomics, is a method of surveying the function of a community of microbes. Previous metatranscriptomics studies focusing on the developing infant gut microbiome have identified characteristics of gene expression of major taxa in the infant microbiome.^19–21^ Observations regarding function and expression help to form a more complete picture of the early microbiome and how important factors may shape it. In this study, we used metatranscriptomics to functionally profile the microbial communities associated with stool samples from the 12-month-old infant gut. In addition to examining how the function of these communities respond to factors such as mode of delivery, breast feeding status and sex of the infant, we also examined how exposure to GDM impacts both microbial community structure and function. To our knowledge, this is the first study to perform metatranscriptomic community functional profiling on the infant gut microbiome in the context of GDM.

## Materials and methods

Detailed methodology can be found in supplemental files.

### Study design

Pregnant women with no prior diagnosis of diabetes were recruited during routine visits in late 2^nd^ to early 3^rd^ trimester at Mount Sinai Hospital, Toronto, Canada. The diagnosis of GDM was made using a sequential 2-step process as per the Diabetes Canada Clinical Guidelines.^11^ In brief, between 24 and 28 weeks of gestation, women undergo a 1 h 50 g glucose challenge test (GCT). Women were recruited both before and after the 1-h 50 g glucose challenge test. The cohort was enriched for mothers that failed the GCT (1 h glucose >7.8 mmol/L) who are more likely to be diagnosed with GDM than those with a passing GCT result. Study participants were thereafter stratified by GDM status as measured through 2 h, 75 g oral glucose tolerance test (OGTT) performed within 1–2 weeks following the GCT, and defined according to established criteria.^11^ The glucose levels sampled during OGTT were used to estimate the area-under-the-glucose-curve (AUC_glucose_) according to the trapezoid rule.

### Data collection

Participating women attended study visits that included questionnaires in late pregnancy and at 3-months and 12-months postpartum. Offspring were assessed at birth, 3, and 12 months. Infants were assessed for anthropometric measurements, and the parent completed questionnaires related to their infant’s health and nutrition. Participating mothers were provided with a sample collection kit to collect their child’s stool sample just prior to the study visit at 3 and 12 months of age. Each kit contained diaper liners, two sterile polypropylene containers, a set of gloves, and plastic collection spoons. Stool samples were collected at home and placed into a sterile polypropylene container. Each stool sample was separated into two containers for 16S rDNA and metatranscriptomic profiling. The samples were kept in the freezer at home and brought with an ice pack at the scheduled visit and were then stored at −80°C. Stool samples were collected and sent for 16S rDNA profiling (3 months and 1 year) and metatranscriptomics profiling (1 year). Additional clinical measurements included: sex, exclusive breastfeeding status, mode of delivery, maternal BMI, and infant birth BMI. Medications and supplements taken by participants during pregnancy include prenatal vitamins, antacids, antihistamines, and medications for hypothyroidism and asthma. Antibiotic and probiotic use, as well as use of other medications was collected postnatally for infants.

### 16S sample preparation and analysis

Prior to nucleotide extraction, stool samples were homogenized. DNA extraction was completed using Omega E.Z.N.A.TM Stool DNA Isolation Kit. Amplification of the V4 region was performed with uniquely barcoded 515F (forward) and 806 R (reverse) sequencing primers to allow for multiplexing.^22^ High-throughput sequencing targeted the hypervariable V4 region of the 16S rRNA gene, using 150 bp x 2 read length. Sequencing was performed at The Centre for the Analysis of Genome Evolution and Function (CAGEF) at the University of Toronto. Primers were adapted to incorporate Illumina adapters with indexing barcodes and sequenced using the Illumina MiSeq platform. QIIME 2 v2023.2 was used to process raw FASTQ files and cluster reads into amplicon sequence variants (ASVs).^23^ Adonis2 from the vegan 2.6–4 R package was used to conduct permutational analysis of variance (PERMANOVA) statistical tests on beta diversity, and ANCOM-BC 2.1.2 was used to test for differential abundance.^24–26^ Analysis of variance (ANOVA) was used to assess for differences in alpha diversity.

### Metatranscriptomics sequencing

Stool collected at 12-months was processed using the RNeasy PowerSoil Total RNA kit, using 400–500 mg of each sample. The extracted RNA was cleaned using the Illumina Ribo-Zero Gold rRNA Removal Kit (Epidemiology), followed by the Zymo RNA Clean and Concentrator kit. The cleaned RNA was then converted into cDNA using the NEBNext Ultra II Directional RNA Library prep kit. Libraries were sequenced using the Illumina NextSeq platform to generate ~ 20,000,000 150bp paired end reads per sample, with 1% PhiX spike-in as standard.

### Metatranscriptomics reads processing

Reads were processed to remove low-quality reads, host reads, and other unnecessary reads using the MetaPro pipeline, which also annotates gene reads using the ChocoPhlAn pangenome database from HUMAnN 2.0 and NCBI non-redundant (NR) protein databases.^27–29^ Human reads were removed through sequence similarity searches against the GRCh38 human reference genome. Enzyme annotations are assigned according to the Swiss-Prot database and taxonomic annotations also utilize ChocoPhlAn and NCBI NR databases, in addition to Kaiju 1.9.0 and Centrifuge 1.0.4.^30–32^

### Metatranscriptomics analyses

adonis from the vegan 2.3–5 R package was used to perform PERMANOVA to compare Bray-Curtis beta diversity between samples.^33^ Data for differential expression was filtered to remove genes with fewer than 6 counts in 6 samples, resulting in 21,563 genes for differential expression and set enrichment analysis. DESeq2 3.16 was used to identify differentially abundant taxa and differentially expressed genes.^34^ Gene set enrichment analysis was done using fgsea 1.26.0.^35^

### Statistical analyses

Maternal and infant descriptive data are presented as the mean ± SE. For comparison between groups, Student’s t test was used for continuous variables and Chi-square for categorical variables. Statistical significance was considered at *p* < .05.

30 total infant gut samples were included in 16S and metatranscriptomics analysis. At the ASV level, 16S alpha diversity comparisons were calculated using ANOVA, and beta diversity comparisons were made using PERMANOVA. ANCOM-BC was used to perform differential taxon abundance analysis.^36^ Bray-Curtis^37^ distance comparisons of gene expression was similarly made using PERMANOVA. Differential gene expression was determined using DESeq2,^34^ and downstream gene set enrichment analysis was performed using the resultant log_2_ fold change values with fgsea.^35^

## Results

### Description of cohort

**Figure 1.**
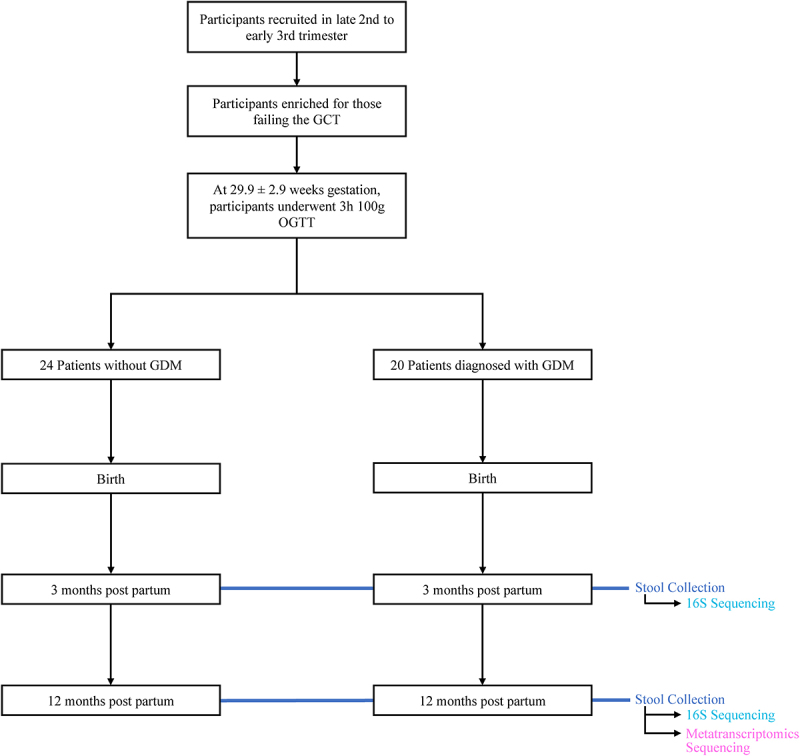
Overview of clinical study design. Patient info was collected at birth, 3 months of infant age, and 12 months of infant age. 16S sequencing was performed on 42 stool samples collected at 3 months and 44 stool samples collected at 12 months. Metatranscriptomics sequencing was performed on 30 of the stool samples collected at 12 months. 28 samples were included for 16S analysis at 3 months. 30 samples were included for 16S and metatranscriptomics analysis at 12 months. See Table 1 for demographic information.

Participants were recruited (*n* = 44) prior to the 2 h OGTT, performed at a mean gestation of 29.9 ± 2.9 weeks. Maternal medication use during pregnancy included prenatal vitamins (*n* = 16), L-thyroxine (*n* = 8), Diclectin (*n* = 3), Iron supplements (*n* = 2), Omega 3 supplements (*n* = 2) and antacids (*n* = 2). Mean gestational age at birth was 39.1 ± 1.2 weeks and mean infant birthweight 3.4 ± 0.5 g. Mothers in this study diagnosed with GDM were either treated with insulin or through diet. None of the women in this study experienced hypertension during pregnancy with the exception of 1 woman with preeclampsia. Of the 44 infants recruited, 30 (*n* = 14 GDM, *n* = 16 nonGDM) had available samples to conduct both 16S rDNA amplicon sequencing and metatranscriptomics at 12 months. Twenty-eight (*n* = 14 GDM, *n* = 14 nonGDM) of these samples were available at 3 months for 16S analysis. The remaining 14 infants who had stool available for only 16S rDNA sequencing and were not included. There was no change in our findings from 16S rDNA sequencing results after exclusion of the 14 infants who did not have stool for metatranscriptome analysis. Postnatally, all infants were exclusively or partially breastfed. Prior antibiotic intake at 3 months of age occurred in one infant (GDM) while probiotics were given to 5 infants (3 nonGDM, 2 GDM). Between 3 and 12 months of age, 3 infants had taken a course of antibiotics (2 nonGDM, 1 GDM) and 3 infants had received probiotics (2 nonGDM, 1 GDM). Participant characteristics are summarized in Table 1 and Supplemental Table 1. Study design is outlined in Figure 1.

**Table 1.** Study demographic table.

		GDM	nonGDM	
	GDM Condition	14	16 (14)	p-value
Average ± SD	Maternal BMI Pre-Pregnancy (kg/m^2^)	25.67 ± 4.46	25.14 ± 5.94	.809
	Weight Gain at the time of the OGTT (kg)	11.01 ± 4.80	13.98 ± 5.38	.0739
	AUC Glucose (mmol × h/L)^a^	17.29 ± 1.69	13.79 ± 1.12	<.0001
	Gestational Age (weeks)	38.63 ± 1.08	39.37 ± 1.12	.0206
	Infant Birth Weight (kgs)	3.40 ± 0.41	3.45 ± 0.52	.707
Number	*Mode of Delivery*			.897
	Vaginal Delivery	10	10 (9)	
	Caesarean-section Delivery	4	6 (5)	
	*Sex*			1.0
	Female	5	6	
	Male	9	10 (8)	
	*Exclusive Breastfeeding* − 3 months^b^			.873
	Exclusive	5	6	
	Partial	9	7	
	*Exclusive Breastfeeding* − 12 months			.847
	Exclusive	3	5	
	Partial	11	11	

^a^The glucose levels sampled during OGTT were used to estimate the area-under-the-glucose-curve.

^b^One 3-month sample did not record breastfeeding status.

Bracketed number indicates number in 3-month infants.

t-test and chi-square test used to determine p-value.

### GDM has limited impact on the microbial community in the developing infant gut

**Figure 2.**
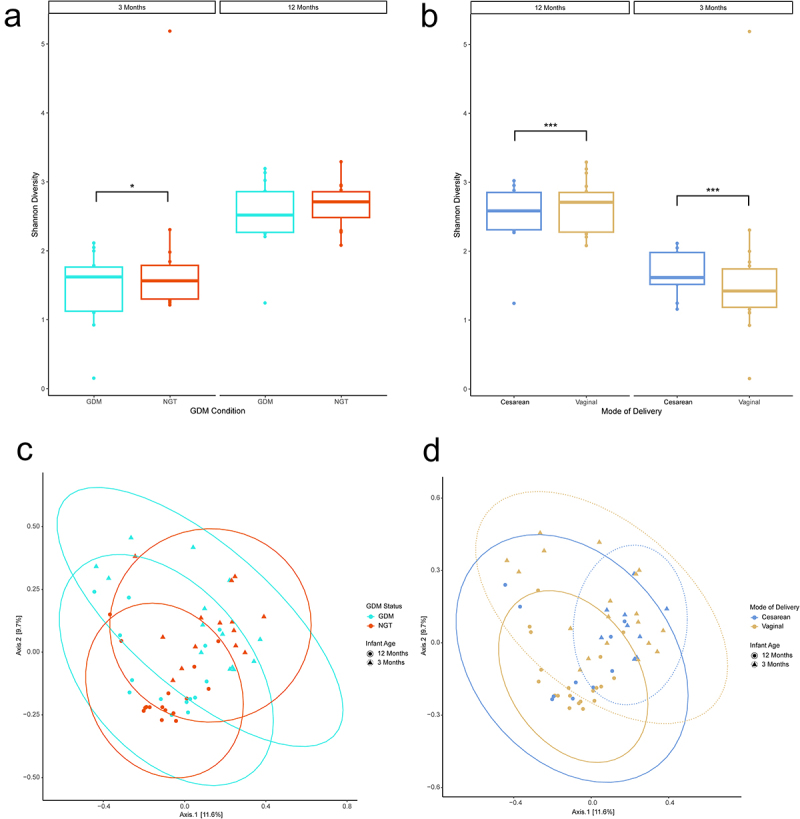
Diversity analyses of 16S data by mode of delivery. (ab) Shannon Alpha diversity analysis. Analysis of Variance (ANOVA) was used to test for statistical significance. (cd) Bray-curtis principal coordinate analysis beta diversity analysis. Ellipses represent 95% confidence interval for a multivariate normal distribution. *n* = 19, Cesarean-section; *n* = 39, Vaginal. *n* = 28, 3 months; *n* = 30, 12 months. *p ≤ .05, **p ≤ .01, ***p ≤ .001.

To examine the impact of GDM on the developing infant gut community, we performed 16S rRNA amplicon sequencing to profile stool samples collected at 3-month (*n* = 28) and 12-month (*n* = 30) timepoints after birth. From the 6,124,664 sequenced reads (median 83,769) we identified 1946 bacterial OTUs across all 58 samples. Shannon Index analysis revealed an increase in alpha diversity for the 12-month samples relative to the 3-month samples (*p* < .001, ANOVA) and that vaginal delivery decreases community diversity at 3 months postpartum (*p* < .001, ANOVA) and increases at 12 months (*p* < .001, ANOVA; Figure 2b). Similarly, nonGDM maternal status infants (*p* = .038; Figure 2a) and exclusive breastfeeding status (EBF; *p* < .001) were associated with an increase in alpha diversity at 3 months, but not at 12 months, in the infant cohort.

Evaluation of beta-diversity (Supplementary Table S2) at the genus level found that infant age (*p* < .001, PERMANOVA), mode of delivery (*p* = .045, PERMANOVA, Figure 2d), and exclusive breastfeeding (*p* = .004, PERMANOVA) had a significant effect on sample clustering. When assessing beta diversity within 3-month samples, only exclusive breastfeeding status had a significant effect on clustering (*p* = .048, PERMANOVA). Neither GDM status nor GDM treatment were associated with any significant clustering effect (Figure 2c, Supplemental Figure 1). Within 12-month samples, none of the comparisons tested had a significant clustering effect on beta diversity. These findings were repeated at the family level. No significant effects were observed at the phylum level.

In order to uncover any taxa-level effects of GDM on gut composition, we examined individual changes in abundance of taxa (Supplementary Table S3). Using ANCOM-BC to test for differential abundance at a genus level, we found that 23 taxa were differentially abundant between the two infant ages, with 17 of these genera decreased in 12-month infants. At 12 months, mode of delivery was associated with 2 differentially abundant genera: *Fusobacterium* (*p* = 3.36e-2, ANCOM-BC), which was more abundant in c-section delivered infants, and *Streptococcus* (*p* = 9.85e-2, ANCOM-BC), more abundant in vaginally delivered infants. At a family level (Supplementary Table S4), Bacteroidaceae was decreased in C-section infants, across all infant ages (*p* = 3.23e-2, ANCOM-BC) and at 3 months (*p* = 4.65e-2, ANCOM-BC).

### Metatranscriptomics taxonomic expression differed significantly from 16S taxonomic composition

To functionally interrogate the gut microbiome of the 12-month-old infants, we performed metatranscriptomics on 30 unique samples, resulting in the generation of an average of 21,154,913.4 150 bp paired-end sequence reads per sample (Supplementary Table S5). After filtering low quality, host, and rRNA, putative mRNA reads were annotated to genes using the MetaPro pipeline.^38^ An average of 25.47% of reads were retained per sample, representing an average of 5,366,805 annotated putative mRNA reads. After gene-level filtering 21,563 genes were retained for analysis, mapping to 607 unique Enzyme Commision (EC) identifiers. This, along with the number of annotated mRNA reads, falls within numbers previously found in metatranscriptomics studies, suggesting adequate gene representation.^26,39–43^ The percentage of putative mRNA reads out of total reads (44.17%) is similar to that established by previous studies.^41,44,45^

**Figure 3.**
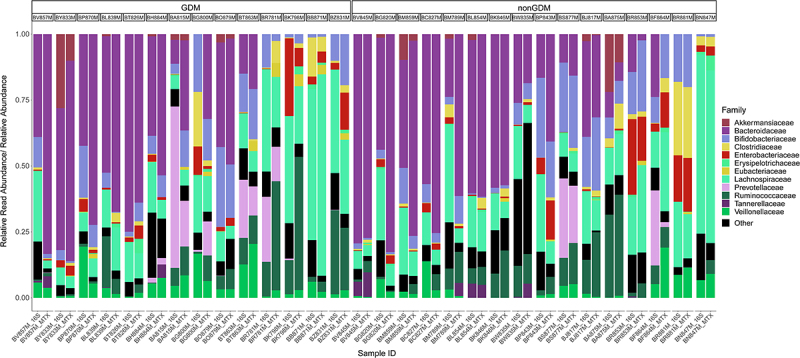
Stacked bar chart illustrating the relative abundance of families in the 16S samples compared to the relative read abundance expressed by each family in metatranscriptomics (MTX) samples. Samples are stratified by gestational diabetes (GDM) condition of the mother. Sorted by decreasing relative abundance of Bacteroidetes phylum in MTX samples. Limited to the top 12 most abundant families. Colours of each family correspond to color of the parent phyla in Supplemental Figure 2 (e.g., Lachnospiraceae is color coded to the Firmicutes phylum). *n* = 30.

The phylum rank was dominated by Bacteroidetes and Firmicutes in both the GDM and nonGDM samples (Supplemental Figure 2). In both GDM and nonGDM samples, the Bacteroidaceae family was most prevalent (Figure 3). We then used DESeq2^34^ to find families that exhibited a significantly different level of expression in metatranscriptomics samples. Of the 404 families detected here, 11 differentially expressing taxa were identified as differentially expressing between GDM and nonGDM infants (Supplemental Table S6), and 6 between vaginally and cesarean-section-delivered infants (Supplemental Table S7). Additionally, Bray-Curtis clustering of gene expression was found to differ significantly from 16S taxonomic composition (*p* < .001) as found by PERMANOVA, further highlighting the difference between expression and composition. This discordance applied to the phylum rank (*p* < .001), as well as the family rank (*p* < .001).

### Mode of delivery has the largest impact on individual gene expression in the 12-month infant gut

**Figure 4.**
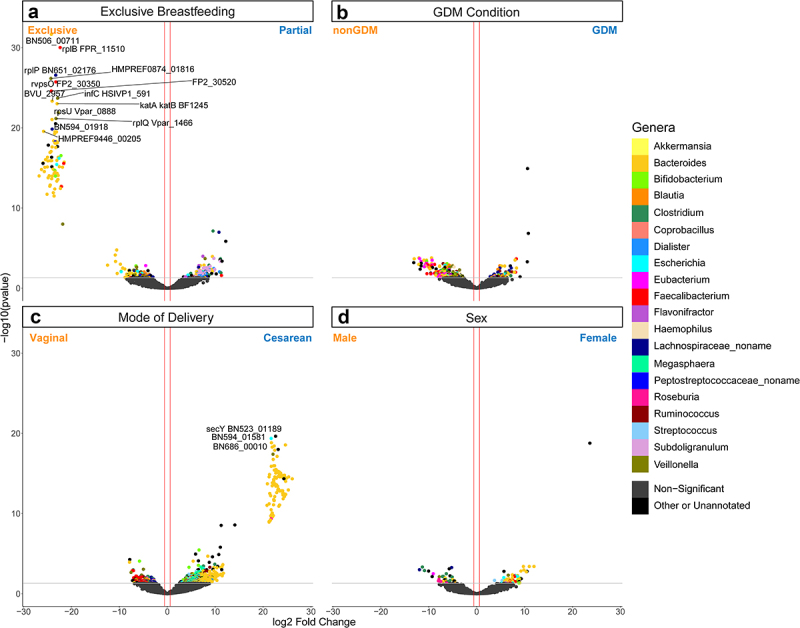
Volcano plot depicting differentially expressed gene (DEG) results for mode of delivery and mode of delivery-controlled factors. Log_2_ fold change and -log_10_(*p* value) are both from results of poscounts DESeq2. Colour of dot corresponds to taxonomic annotation. Grey points are non-DEGs. (a) nonGDM vs GDM. (b) Vaginal birth vs cesarean section birth. (c) Male vs female. (d) Exclusive breastfeeding vs. partial breastfeeding. Upregulation and downregulation are relative to the labels. n = 21563 genes.

We next examined gene-level expression in our data. DESeq2 identified 980 DEGs associated mode of delivery, nearly thrice the number of DEGs found in any other comparison (Figure 4c, Supplemental Figure S3a-d, Supplemental Table S8). Considering the larger impact of mode of delivery on gene expression, each comparison was then reexamined after controlling for the impact of mode of delivery on function. Here, 226 genes were differentially expressed in the GDM status condition, 90 DEGs associated with sex, and 269 DEGs linked to EBF (Figure 4a,b,d). Examination of the genera that express these DEGs reveals that *Bacteroides* are usually the most abundant taxa, with the exception of GDM condition-associated DEGs, where *Veillonella* expressed 20% of the genes. *Faecalibacterium*-annotated genes are also common across these DEGs. In EBF-associated DEGs, a significantly higher number of DEGs were annotated to *Subdoligranulum* than might be expected in the population (*p* < .001, hypergeometric test).

### Gestational diabetes condition was not associated with any enriched gene ontology terms

**Figure 5.**
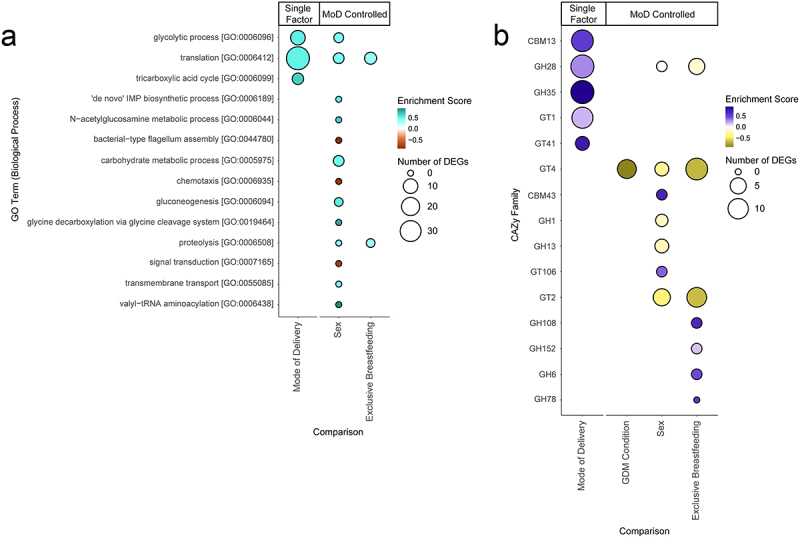
Bubble charts detailing each Gene Ontology (GO) biological process term and carbohydrate-active enzyme (CAZy) family that was significantly enriched in our differential expression results. Enriched terms were identified using the fgsea R package.^35^ Size of bubble indicates the number of DEGs from a given functional category associated with the factor. Enrichment score indicates degree and direction of enrichment for a given term. GO terms and CAZy families are sorted by adjusted p-value. Mode of delivery (MoD) controlled terms are indicated. (a) Bubble chart of GO term enrichment. MoD-controlled GDM condition is omitted as there were no significant terms. (b) Bubble chart of CAZy enzymes. CBM = Carbohydrate-Binding Modules, GH = Glycoside Hydrolases, GT = GlycosylTransferases.

In order to examine which broader categories of function were represented by these DEGs, we performed a gene set enrichment analysis using the fgsea R package.^35^ Here, we examined enrichment using three different databases: the biological process ontology of the gene ontology resource (GO; Figure 5a), the comprehensive antibiotic resistance database (CARD; Supplemental Figure 4), and the carbohydrate-active enzymes database (CAZy/CAZyme; Figure 5b).^46–48^ Mode of delivery was associated with an increase of tricarboxylic acid cycle (TCA; P 2.46e-2, fgsea), glycolytic process (*p* = 2.46e-2, fgsea), and translation (*p* < 0.001, fgsea) GO terms in C-section infants. Similarly, in the CAZy enrichment, mode of delivery was associated with 5 CAZy families, all of which upregulated in C-section infants as well.

After controlling for mode of delivery, GDM DEGs were enriched for the glycosyl transferase (GT) family 4 CAZyme (*p* = 9.03e-4, fgsea) within GDM infants. EBF DEGs were enriched for 7 CAZy families, including 5 glycoside hydrolase (GH) families, and two CARD sets, all of which are increased in enrichment in partially breastfed infants, apart from an isoniazid resistance term (*p* = 4.26e-2, fgsea). Significant GO terms include translation (*p* = 5.88e-3, fgsea) and proteolysis (*p* = 3.58e-7, fgsea), both of which are enriched in partially breastfed infants. Sex was associated with the highest number of GO terms and CAZy families, though direction of enrichment was not consistent. These include 5 carbohydrate metabolism terms enriched in female infants, and 2 motility terms enriched in male infants. Terms relating to microbiome product usage or generation such as short-chain fatty acids and LPS were not found to be enriched in any comparison.

## Discussion

This case control study sought to profile the changes experienced by the infant gut microbiome in response to a pregnancy complicated by GDM, while concurrently exploring the effect of mode of delivery, breastfeeding, and sex. While previous studies have examined the impact of GDM on the composition of the infant gut microbiome, we believe this is the first study that incorporates a functional metatranscriptomics approach. Our results indicate that maternal GDM is associated with a decrease in alpha diversity across all infant ages and in the 3-month infants specifically. This corroborates existing abundance-based microbiome research, in which alpha diversity has been shown to be significantly lower in neonates of mothers with GDM.^49–51^ This is not always consistent in literature; studies involving infants ranging from 1-week to 4-years of age have found no differences in alpha diversity between GDM and nonGDM infants.^17,52^ As GDM did not have a significant impact on alpha diversity in the 12-month infants, it is possible that alpha diversity in GDM infants increases to meet the levels of nonGDM infants sometime before 12 months of age due to changing diet and environmental exposure.^3^ This may also apply to the non-significant difference in beta diversity between GDM and nonGDM infants in our data. Previous literature indeed have found differences in beta diversity in GDM infants.^16,17,51,52^ In our data, it is also possible that the nonGDM mothers having already failed the GCT indicating some degree of dysglycemia resulted in the interindividual differences between GDM and nonGDM infant guts failing to be distinct enough. Furthermore, the women with GDM participating in our study were extremely well controlled with diet and/or insulin, and this may further have diminished differences in infant microbiota, as evidenced by similar birth weights between GDM and nonGDM infants (Table 1).

Mode of delivery was linked with a significant difference in alpha diversity in 3-month and 12-month infants. Mode of delivery also significantly associated with beta diversity when examining samples of all ages. Existing literature offers somewhat conflicting results in regards to how mode of delivery induces compositional changes to the microbiome over the first year of life.^53–57^ While mode of delivery was associated with changes in the gut microbiome early on, some studies suggest its influence wanes by 6 months of age, possibly as early as 8 weeks.^57–61^ Research centered on the gut microbiome of older children (≥3 years) have found few differences in overall diversity associated with birthing method, though individual taxa had changed.^62,63^ Our results support the notion that perturbations to alpha and beta diversity by delivery remain detectable within the first year.

In our 16S samples, we found a reduction of Bacteroidaceae abundance in C-section infants across both infant ages and at 3 months specifically. This has previously been shown to be a common result of C-section infants receiving less exposure to the maternal microbiome.^60,64^ In comparison, 6 families were differentially expressive in the metatranscriptomics samples, which also include a reduction in Lactobacillaceae- and Ruminococcaceae-derived transcripts in C-section delivered infants. Both of these families contains species considered beneficial to the infant gut, such as *Lactobacillus acidophilus* and *Faecalibacterium prausnitzii*.^65–69^ These taxa were likely passed down to the vaginally delivered infants,^42,70^ and the reduction in abundance of these species could represent a long-term consequence of C-section delivery.

Since there were no mothers in this study that exclusively used infant formula milk, groups compared between exclusive and partial breastfeeding diets. Therefore, the effect being measured is likely more subtle than comparison between exclusively breastfed and exclusively formula-fed infant gut microbiota. Breast milk aids in nurturing infant gut microbes through a supply of human milk oligosaccharides (HMOs) among other metabolites, vitamins and immunoglobulins.^66,71^ These HMOs are utilized by *Bifidobacterium* and *Bacteroides* species in the gut,^72^ the latter of which expressed a large proportion of transcripts in our data. A meta-analysis of 7 studies conducted by Ho et al. found that non-exclusively breastfed infants under 6 months of age displayed higher gut Shannon alpha diversity.^73^ Breastfeeding status also had a strong effect on our 16S data, with partially breastfed infants experiencing a decrease in alpha diversity across all infant ages and at 3 months. Similarly, breastfeeding status was associated with significant clustering by beta-diversity at all infant ages and at 3 months; it is possible these differences in diversity are eventually lost by 12 months. Increasing variety in diet, such as introduction of solid foods, or changes in milk feeding frequency may contribute to this increase in diversity.^74,75^

Few studies on the infant gut microbiome have applied metatranscriptomics to study the infant gut microbiome, and they have tended to focus on preterm births.^26,76,77^ Beyond these, one study reported pathway changes in a longitudinal study over the first year of life,^19^ while another study profiled changes in microbiome activity alongside solid food adaptation, outlining steps toward an adult-like composition.^21^ In comparison, our metatranscriptomics results focused on twelve-month old infants, aiming to reveal factors that may have sustained effects on microbiome activity. Our results demonstrate that by 12 months, the persisting effect of maternal GDM status and EBF is modest, while mode of delivery and sex both influence the function of the microbiome, based on functional enrichment analysis.

As there have been no metatranscriptomics studies on GDM, most predictions on the functional changes GDM induce are derived from correlating taxonomic abundance changes to factors in health.^50,78^ We found no significantly enriched functions, as defined by GO terms, even after controlling for the effects of mode of delivery. We found only one significantly enriched CAZy family (GT4) upregulated in GDM infants after controlling for mode of delivery, despite GDM being primarily an issue of carbohydrate metabolism in the host. Notably, GTs have previously been implicated in diabetes as a potential therapeutic target^79^ and have been observed to change in activity in response to hyperglycemia.^80^ However, the higher number of CAZymes enriched in our other analyses suggest a marginal influence on carbohydrate metabolism. While there were a higher number of DEGs associated with GDM than sex, our results suggest that, by 12 months of age, there is no significant persistent perturbation of functional categories in the infant gut resulting from maternal GDM status. As highlighted below, we cannot rule out the possibility that the diet and/or treatment with insulin of the mothers diagnosed with GDM in this study were able to overcome any potential negative effects of GDM on the infant gut, which has previously been shown through metagenomics by Sugino et al.^81^ The study compared a conventional diet with a diet that was higher in complex carbohydrates and lower in fat in women diagnosed with GDM. As a result, infants of the mothers fed the treatment diet displayed increased gut alpha diversity over time. Indeed, previous work from our cohort has consistently demonstrated lower birthweight of infants born to women with GDM, compared to those with normal or milder dysglycemia during pregnancy, which indicates excellent glycemic control in this GDM cohort during pregnancy.^82^

Three terms were significantly enriched in infants delivered through C-section: translation, glycolytic process, and tricarboxylic acid cycle. The enrichment of two carbohydrate metabolism-related terms together with translation, which correlates with cell growth,^83^ suggest that there may be a relative increase in microbial cell proliferation in infants delivered through C-section compared to vaginally delivered infants. This is supported by the overall direction of expression for most mode of delivery DEGs (Figure 4b) and the direction of the significantly enriched CAZy families (Figure 5b), all of which are enriched in samples associated with C-sections. Along with the high number of DEGs and the observed changes in diversity, our results point to the mode of delivery as having a clear effect on both the structure and function of the 12-month-old infant gut microbiome.

Post mode of delivery-control, sex was associated with 13 enriched GO terms, the most of any comparison tested. Five terms were directly related to nutrient metabolism, primarily carbohydrate metabolism, all of which had been enriched within female infant gut microbiomes. Similarly, seven CAZy families were significantly enriched, though these were not conformally unidirectional. PICRUSt analysis of 16S data has previously identified some sex-specific differences between pre-term, dizygotic twin male and female gut function; though there were carbohydrate metabolism pathways differentially regulated in these results, many other unrelated pathways were differentially regulated as well.^24^ Hormones appear to affect the composition of the infant gut since birth,^25^ and our results point to some early microbiome differences in function as well.

Only two GO terms – proteolysis and translation – were enriched in the mode of delivery-controlled EBF. A previous meta-analysis by Ho et al.^73^ involving infants less than six months of age found that most KEGG pathways that differed between partially and exclusively breastfed infants were involved with metabolism. One previous metatranscriptomics study in preterm infants found that breastfed infants and formula-fed infants were enriched for different metabolic functions.^26^ Though in our data, there were seven CAZy families significantly enriched between infants of varying EBF status, they were not consistently enriched in one direction.

Several additional methodological considerations in this study warrant further discussion. Firstly, the infants of mothers with GDM were monitored during pregnancy in a tertiary care center, and their infants demonstrate lower birthweight than the control group, suggesting effective glucose management during pregnancy.^82^ As a result, this may have acted to mitigate the potential microbiome-modifying effects of the condition. Secondly, the reference control group was composed of mothers who failed the initial GCT but did not meet criteria for GDM on glucose tolerance testing. While glycemic management intervention is therefore not indicated for this group of mothers, they are experiencing subclinical dysglycemia. The two groups of mothers were not significant dissimilar in terms of BMI, nor did their infants separate based on birth weight. While there have been several similar cohort studies in which this lack of group body habitus divergence was observed,^16,51,84^ we must consider the alternate possibility that even exposure to subclinical dysglycemia may alter the infant gut microbiome in a manner which is similar to GDM. Although prenatal medication was recorded, perinatal and postnatal medications were not, both of which have been observed to impact the infant microbiome.^85^ Furthermore, the relatively small sample size together with the relatively late (12 months) timepoint used in this study may have limited our ability to identify functional correlates of GDM status in the mother, which might be expected to be more prominent at timepoints closer to birth. Previous benchmarking studies have found that different methods applied at each step of sample collection, processing (e.g. choice of method for nucleic acid extraction^86,87^ and analysis can bias the recovery of nucleotides from individual taxa. As a result, certain microbes may be underrepresented in this study, although a previous study suggests that biases may be reduced for RNA extraction protocols.^88^ Lastly, as this study was exploratory and metatranscriptomics has not been previously applied to studying GDM to our knowledge, there were no prior studies for sample size calculations. However, previous studies involving 16S analysis of GDM newborns and metatranscriptomics analysis of infants examining breastfeeding modes have similar sample sizes,^16,26^ and the Bray-Curtis analysis utilized in this study has been shown to be the most sensitive indicator of beta diversity.^89^

While GDM has been observed to affect the composition of the early infant microbiome, in this study we were able to elucidate the functional consequences of GDM further into the development of the infant gut. Though our results support some changes in alpha diversity, GDM exerted little effect on function in the 12-month-old infant gut. As the gut develops with the introduction of solid foods and weaning from breastfeeding, the effect of GDM exposure becomes less prominent against an increasingly complex gut community. Similarly, while there may be differences in diversity remaining, our results suggest that the impact of EBF wanes by one year. Some functional differences are visible at 12 months in sex and mode of delivery, with the latter likely having the greatest influence. These data may indicate that, while mode of delivery appears to impact function and diversity for longer than anticipated, GDM may not have persistent effects on the function nor composition of the infant gut microbiome, at least when compared to mothers who failed OCT but did not experience GDM. Regulation of diet and glycemic control may alleviate the potential impact of GDM on the infant gut microbiome. Future studies in this area could explore correlations between the maternal and infant gut, the mitigating effects of diet, peripartum antibiotics, and other interventions on the impact of GDM on the infant gut.

## Supplementary Material

Supplemental Material

## Data Availability

Sequence data is deposited at the NCBI under BioProject repository identifier PRJNA1013505.
