# Supplementary material for: The impact of gestational diabetes on functional capacity of the infant gut microbiome is modest and transient
Source: Gut Microbes. 2024 May 26;16(1):2356277. doi: 10.1080/19490976.2024.2356277 (PMC11135868; doi:10.1080/19490976.2024.2356277)
Supplement: Supplemental Material [file KGMI_A_2356277_SM9103.zip › gapkids_supplemental_appendix.docx]

**SUPPLEMENTARY APPENDIX**

**SUPPLEMENTARY TABLES AND FIGURES**

Supplementary Table 1. Study Demographic Table by Infant Age and Data

|  |  | 16S |  | Metatranscriptomics |
| --- | --- | --- | --- | --- |
|  |  | 3 Months | 12 Months | 12 Months |
| Maternal Glucose Status | GDM | 14 | 14 | 14 |
|  | nonGDM | 14 | 16 | 16 |
| Mode of Delivery | Vaginal | 19 | 20 | 20 |
|  | Caesarean Section | 9 | 10 | 10 |
| Sex | Female | 11 | 11 | 11 |
|  | Male | 17 | 19 | 19 |
| EBF | Exclusive | 11^1^ | 8^2^ | 8 |
|  | Partial | 16 | 22 | 22 |
| Total Samples |  | 28 | 30 | 30 |

^1^ One 3-month sample did not record breastfeeding status

^2^ 12-month old infants were fed a varied solid diet, but their source of milk has exclusively been breastfeeding

Supplementary Table 2. Beta-Diversity PERMANOVA Results

|  | All Infant Ages (p-values) | 3 Months (p-values) | 12 Months (p-values) |
| --- | --- | --- | --- |
| Visit | 0.001 | - | - |
| GDM Condition | 0.713 | 0.991 | 0.559 |
| Mode of Delivery | 0.069 | 0.105 | 0.485 |
| Sex | 0.695 | 0.952 | 0.322 |
| EBF | 0.008 | 0.039 | 0.634 |
| Gestational Age | 0.390 | 0.369 | 0.618 |
| Infant Birth BMI-z | 0.403 | 0.341 | 0.424 |

Supplementary Table 3. ANCOM-BC Number of Differentially Abundant Genera

|  | All Infant Ages | 3 Months | 12 Months |
| --- | --- | --- | --- |
| Visit | 25 | - | - |
| GDM Condition | 0 | 0 | 0 |
| Mode of Delivery | 0 | 0 | 2 |
| Sex | 0 | 0 | 0 |
| EBF | 0 | 0 | 0 |

Supplementary Table 4. ANCOM-BC Number of Differentially Abundant Families

|  | All Infant Ages | 3 Months | 12 Months |
| --- | --- | --- | --- |
| Visit | 11 | - | - |
| GDM Condition | 0 | 0 | 0 |
| Mode of Delivery | 1 | 1 | 0 |
| Sex | 0 | 0 | 0 |
| EBF | 0 | 0 | 0 |

Supplementary Table 5. MetaPro Pipeline Metrics

|  | Total Reads | % high quality reads | % host reads | % rRNA + tRNA reads | % putative mRNA reads | % annotated putative mRNA | # of annotated mRNA reads |
| --- | --- | --- | --- | --- | --- | --- | --- |
| Mean | 2.12e8 | 97.832 | 0.115 | 53.377 | 44.152 | 60.781 | 5.37e8 |
| SD | 8.32e7 | 0.496 | 0.186 | 18.308 | 18.194 | 11.514 | 2.98e8 |

Supplemental Table 6: DESeq2 Number of Differentially Abundant Families in GDM

| Family | log_2_FoldChange | padj |
| --- | --- | --- |
| Acidaminococcaceae | 5.871504953 | 2.46E-06 |
| Rikenellaceae | -5.362586702 | 7.85E-06 |
| Prevotellaceae | 4.565009262 | 1.46E-05 |
| Synechococcaceae | 6.133206138 | 0.000347 |
| Morganellaceae | 4.025327881 | 0.000956 |
| Tannerellaceae | -3.259568985 | 0.0044 |
| Oscillospiraceae | -2.932605088 | 0.007054 |
| Eubacteriaceae | 1.705293264 | 0.007899 |
| Thermoanaerobacteraceae | 2.465265978 | 0.012009 |
| Odoribacteraceae | 2.676915271 | 0.0304 |
| Pasteurellaceae | 2.107444975 | 0.045877 |

Supplemental Table 7: DESeq2 Number of Differentially Abundant Families in Mode of Delivery

| Family | log2FoldChange | padj |
| --- | --- | --- |
| Rikenellaceae | 3.843458 | 0.005791 |
| Planococcaceae | -3.33076 | 0.001878 |
| Lactobacillaceae | 2.91745 | 0.00234 |
| Ruminococcaceae | -1.59875 | 0.030911 |
| Fusobacteriaceae | -2.42508 | 0.02779 |
| Chromobacteriaceae | -7.82982 | 6.90E-05 |

Supplementary Table 8. DESeq2 Number of Differentially Expressed Genes

|  | Single Factor | Mode of Delivery Controlled |
| --- | --- | --- |
| GDM Condition | 294 | 226 |
| Mode of Delivery | 980 | - |
| Sex | 260 | 90 |
| EBF | 342 | 269 |

**Supplemental Figure 1**

Bray-Curtis Principal Coordinate Analysis Beta Diversity analysis. Ellipses represent 95% confidence interval for a multivariate normal distribution. n = 20, Diet; n = 8, Insulin; n = 30, No GDM. n = 28, 3 months; n = 30, 12 months.

**Supplemental Figure 2.**

Stacked bar chart illustrating the relative abundance of phyla in the 16S samples compared to the relative read abundance expressed by each family in metatranscriptomics (MTX) samples. Samples are stratified by gestational diabetes (GDM) condition of the mother. Sorted by decreasing relative abundance of Bacteroidetes phylum in MTX samples. Limited to the top 7 most abundant phyla. Colours of each phylum correspond to colour of the child family in Figure 3. n = 30.

**Supplemental Figure 3.**

Volcano plot depicting differentially expressed gene (DEG) results for single factors. Log_2_ fold change and -log_10_(p value) are both from results of poscounts DESeq2. Blue points indicate DEGs that are upregulated in a condition. Red points indicate DEGs that are downregulated in a condition. Grey points are non-DEGs. (A) Exclusive breastfeeding vs. partial breastfeeding. (B) nonGDM vs GDM. (C) Vaginal birth vs caesarean section birth. (D) Male vs female. Upregulation and downregulation are relative to the labels. n = 21563 genes.

**Supplemental Figure 4.**

Bubble charts detailing each Comprehensive Antibiotic Resistance Database (CARD) resistance gene family that was significantly enriched in our differential expression results. Enriched terms were identified using the fgsea R package^32^. Size of bubble indicates the number of DEGs from a given gene family associated with the factor. Enrichment score indicates degree and direction of enrichment for a given term. CARD resistance genes are sorted by adjusted p-value.

**SUPPLEMENTARY METHODS**

**Study Design**

Participating women attended study visits that included nurse-administered questionnaires for personal medical, obstetrical, and family history, anthropometric measurement, and tests of lipid profile, insulin sensitivity, glucose tolerance, and adipokines in late pregnancy and at 3-months and 12-months postpartum.

The exclusion criteria for this study were as follows:

- Infants born less than 37 weeks gestation or greater than 42 weeks of gestation
- Multiparity
- Infants with significant medical illness requiring prolonged or repeated hospitalization
- Infants with medical conditions or taking medications (e.g. glucocorticoids) known to alter cardiometabolic risk
- Stool samples of infants who had antibiotics and/or probiotics within 3 months of collection were also excluded from microbiome analysis

Data was collected from the chart and by questionnaires for the following:

- Parental BMI
- Maternal pre-gravid body mass index
- Weight gain during pregnancy
- Mode of delivery
- Infant feeding history (exclusive breastmilk vs formula and breast milk; age of introductions of solids)
- Infant Sex
- Infant Birth Weight
- Gestational Age at Birth
- Other factors that may affect microbiome (ex. Antibiotic, probiotic use)

**16S Sample Preparation and Analysis**

Amplification reactions were performed with 12.5 µL of KAPA2G Robust HotStart ReadyMix. This step was carried out in triplicate to reduce amplification bias. DNA quality was assessed spectrophotometrically. QIIME 2 v2023.2 was used to process raw FASTQ files, remove low-quality reads, and assemble and merge paired reads^84^. Deblur v1.1.1 was used to cluster reads into ASVs^94^. Samples were visualized using phyloseq 1.44.0 and ggplot 3.4.1^95,96^.

**Metatranscriptomics Sequencing**

Metatranscriptomic sequencing was performed on only 12-month samples. Changes to the kit protocol include an incubation with SR4 at 1 hour at room temperature at step 9, heat pellet at 45°C for 10 minutes at step 12, incubation with SR4 overnight at -20°C at step 17, and elution in 100 μL of SR7. DNA was removed from the sample by incubating 44 μL of RNA with 1 μL of Turbo DNase and 5 μL of Turbo 10X Buffer at 37°C for 30 minutes. RNA was cleaned with Zymo RNA Clean and Concentrator and processed by BioAnalyze for quality and quantity of RNA. 2.5 μg was added to RiboZero Gold kit to remove rRNA^97^. cDNA was amplified using the NEBNext Ultra II Directional RNA Library prep kit, following protocol for Ribosome Depleted RNA. Qubit/ PicoGreen was applied to the barcoded cDNA libraries, and the final library was processed by BioAnalyze on DNA chip to determine size. The samples were amplified using barcoded adapters and 15x cycles of PCR, as suggested in the manual based on the input RNA concentration , and sequenced on the Illumina NextSeq platform to generate ~20,000,000 150bp paired end reads per sample, with 1% PhiX spike-in as standard.

**Metatranscriptomics Reads Processing**

Low-quality reads are defined with a quality score below 75 according to FastQC 0.11.9^22^. Adapter sequences are identified and removed using AdapterRemoval v2.1.7 and VSEARCH v2.7.1^98,99^. pBLAT 2.0 and BWA 0.7.17 were used to filter contaminants and vectors using the UniVec_Core dataset^90,100,101^. Host reads corresponding to the GRCh38 human reference genome were removed followed by use of BARRNAP v0.9 and Infernal to remove rRNA sequences^102,103^. Duplicate reads that were previously removed are added back in, and the mRNA is then assembled into contigs using the rnaSPAdes v3.14.1 transcript assembly algorithm and MetaGeneMark v1 to annotate contigs to putative genes^104,105^. Contigs are then annotated to genes and assigned taxonomic information and enzyme function. Metapro utilizes three sequence similarity search tools including BWA 0.7.17, pBLAT 2.0, and DIAMOND 0.9.19^100,101,106^. Enzyme annotation is carried out using DETECT v2, PRIAM version 2018, and DIAMOND 0.9.19 ^106–108^.

**Metatranscriptomics Analyses**

DESeq2 differential expression analysis was performed with the poscounts size factor estimator. CAZy annotations were retrieved using dbCAN3^109^. CARD annotations were retrieved using CARD’s Resistance Gene Identifier^34^. Visualizations of figures 2-5 and supplemental figures 1-3 were done through ggplot 3.4.1^96^.

**REFERENCES**

94. Deblur Rapidly Resolves Single-Nucleotide Community Sequence Patterns | mSystems. Accessed September 12, 2023. https://journals.asm.org/doi/full/10.1128/msystems.00191-16

95. McMurdie PJ, Holmes S. phyloseq: An R Package for Reproducible Interactive Analysis and Graphics of Microbiome Census Data. *PLOS ONE*. 2013;8(4):e61217. doi:10.1371/journal.pone.0061217

96. Wickham H. *Ggplot2: Elegant Graphics for Data Analysis*. 2nd ed. 2016. Springer International Publishing : Imprint: Springer; 2016. doi:10.1007/978-3-319-24277-4

97. Benes V, Blake J, Doyle K. Ribo-Zero Gold Kit: improved RNA-seq results after removal of cytoplasmic and mitochondrial ribosomal RNA. *Nat Methods*. 2011;8(11):iii-iv. doi:10.1038/nmeth.f.352

98. Schubert M, Lindgreen S, Orlando L. AdapterRemoval v2: rapid adapter trimming, identification, and read merging. *BMC Res Notes*. 2016;9(1):88. doi:10.1186/s13104-016-1900-2

99. Rognes T, Flouri T, Nichols B, Quince C, Mahé F. VSEARCH: a versatile open source tool for metagenomics. *PeerJ*. 2016;4:e2584. doi:10.7717/peerj.2584

100. Kent WJ. BLAT--the BLAST-like alignment tool. *Genome Res*. 2002;12(4):656-664. doi:10.1101/gr.229202

101. Li H, Durbin R. Fast and accurate short read alignment with Burrows-Wheeler transform. *Bioinformatics*. 2009;25(14):1754-1760. doi:10.1093/bioinformatics/btp324

102. Seemann T. BARRNAP. Published online August 25, 2018.

103. Nawrocki EP, Eddy SR. Infernal 1.1: 100-fold faster RNA homology searches. *Bioinformatics*. 2013;29(22):2933-2935. doi:10.1093/bioinformatics/btt509

104. Bushmanova E, Antipov D, Lapidus A, Prjibelski AD. rnaSPAdes: a de novo transcriptome assembler and its application to RNA-Seq data. *GigaScience*. 2019;8(9):giz100. doi:10.1093/gigascience/giz100

105. Zhu W, Lomsadze A, Borodovsky M. Ab initio gene identification in metagenomic sequences. *Nucleic Acids Res*. 2010;38(12):e132. doi:10.1093/nar/gkq275

106. Buchfink B, Xie C, Huson DH. Fast and sensitive protein alignment using DIAMOND. *Nat Methods*. 2015;12(1):59-60. doi:10.1038/nmeth.3176

107. Nursimulu N, Xu LL, Wasmuth JD, Krukov I, Parkinson J. Improved enzyme annotation with EC-specific cutoffs using DETECT v2. *Bioinformatics*. 2018;34(19):3393-3395. doi:10.1093/bioinformatics/bty368

108. Claudel-Renard C, Chevalet C, Faraut T, Kahn D. Enzyme-specific profiles for genome annotation: PRIAM. *Nucleic Acids Res*. 2003;31(22):6633-6639. doi:10.1093/nar/gkg847

109. Zheng J, Ge Q, Yan Y, Zhang X, Huang L, Yin Y. dbCAN3: automated carbohydrate-active enzyme and substrate annotation. *Nucleic Acids Research*. 2023;51(W1):W115-W121. doi:10.1093/nar/gkad328
