# Supplementary figures and images for: The impact of gestational diabetes on functional capacity of the infant gut microbiome is modest and transient
Source: Gut Microbes. 2024 May 26;16(1):2356277. doi: 10.1080/19490976.2024.2356277 (PMC11135868; doi:10.1080/19490976.2024.2356277)

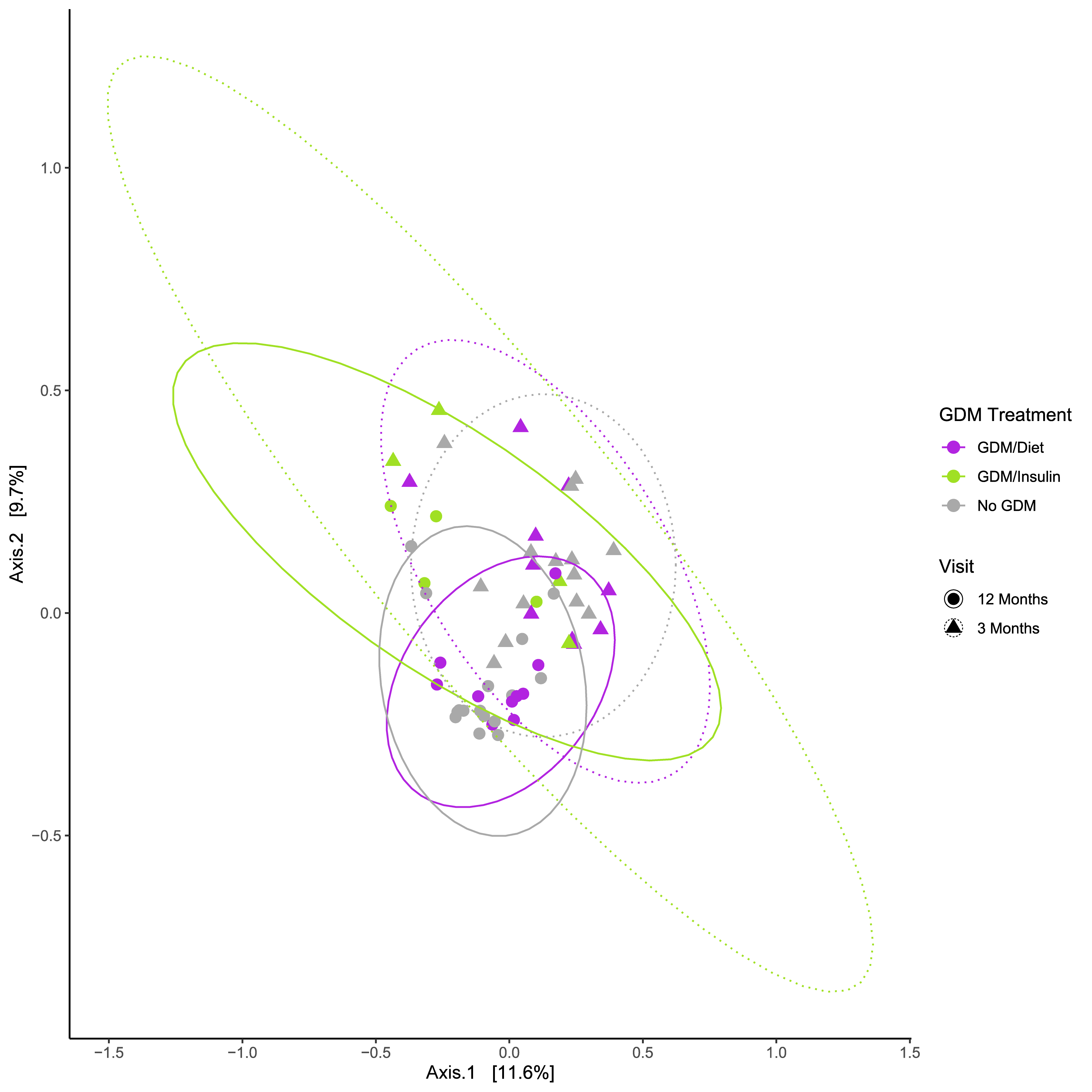

Supplement: Supplemental Material [file KGMI_A_2356277_SM9103.zip › gapkids supplemental figure 01.tif]

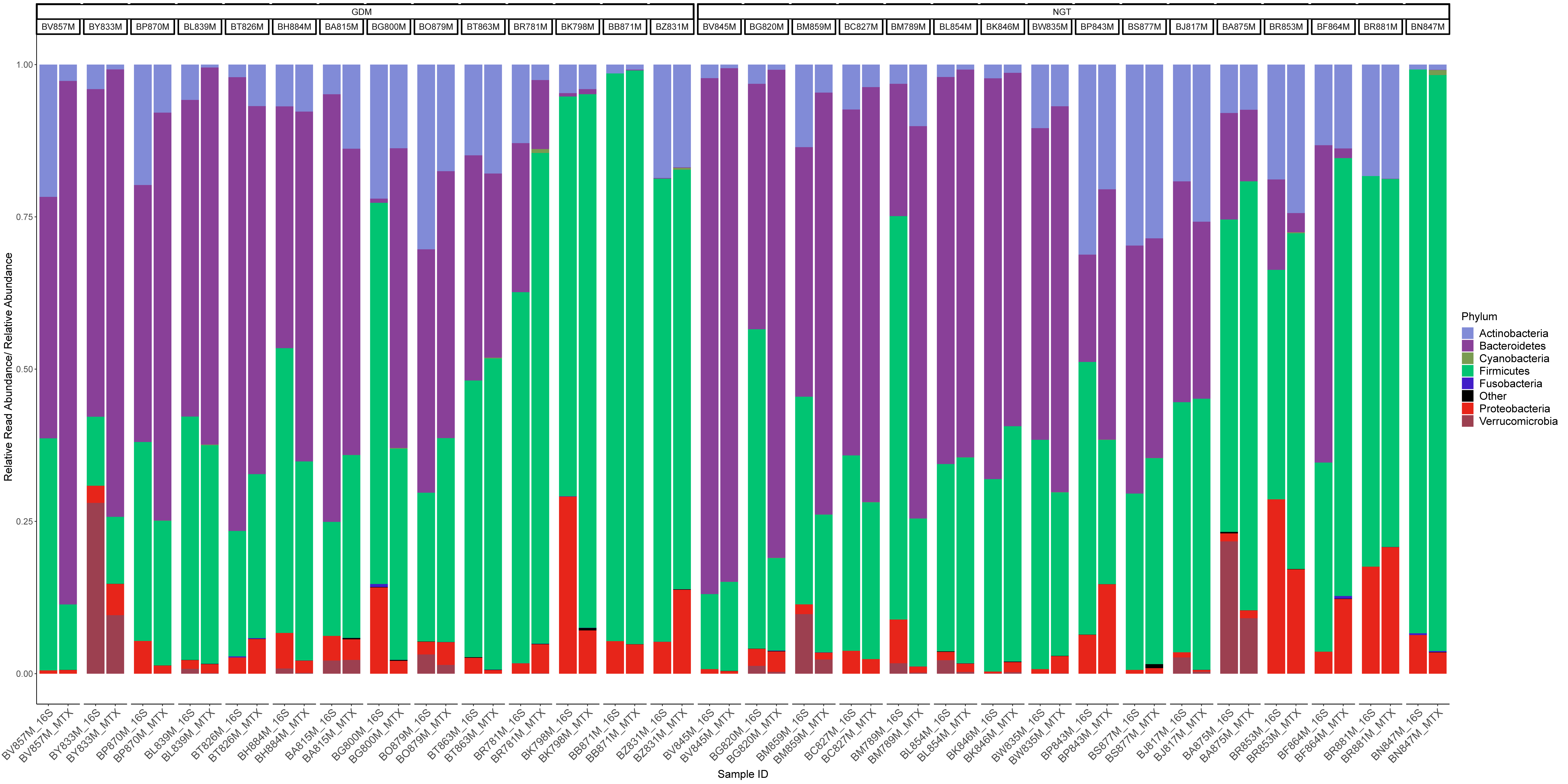

Supplement: Supplemental Material [file KGMI_A_2356277_SM9103.zip › gapkids supplemental figure 02.tif]

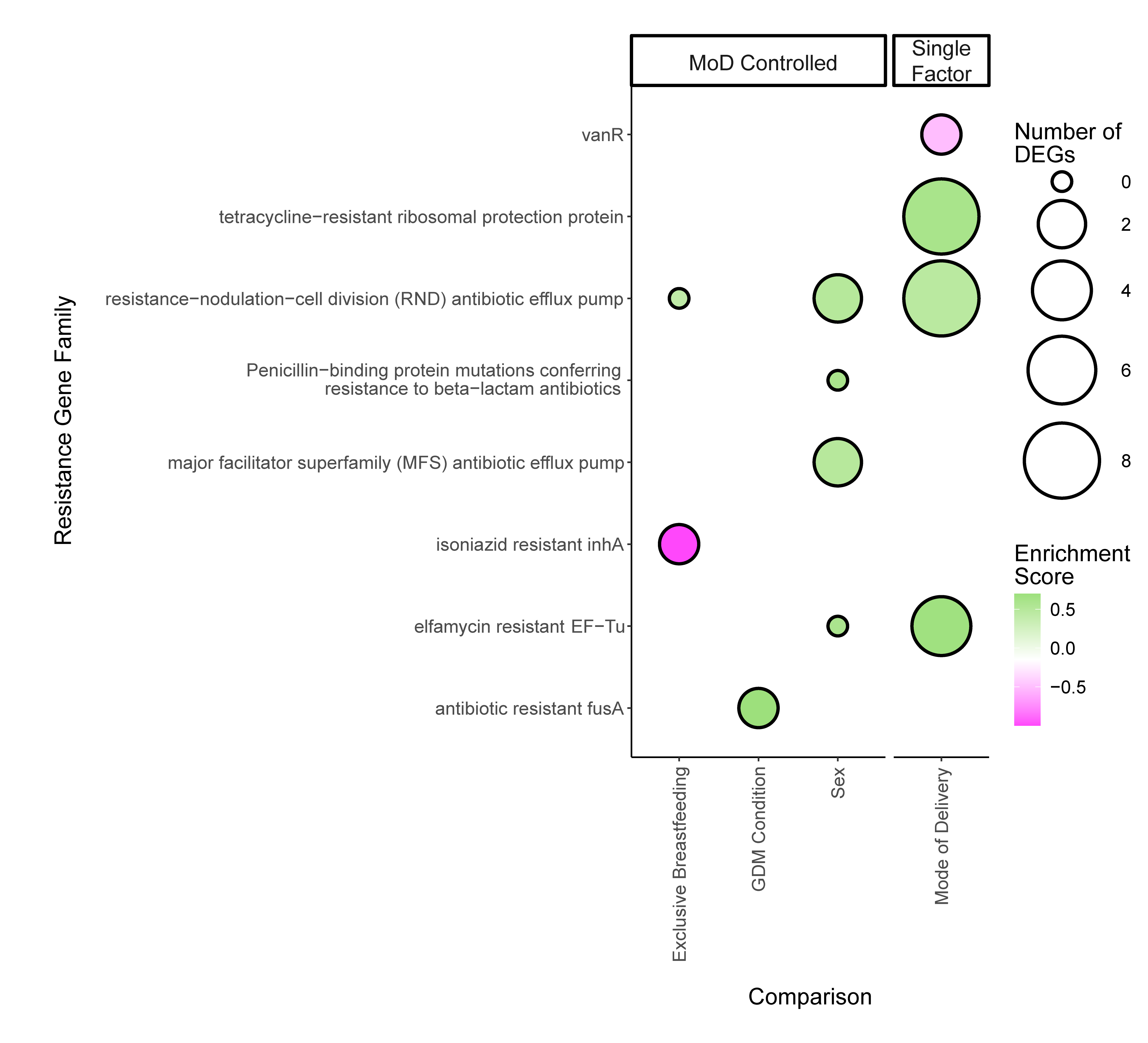

Supplement: Supplemental Material [file KGMI_A_2356277_SM9103.zip › gapkids supplemental figure 04.tif]
